# The ulcerative colitis endoscopic index of severity score is superior to reflecting long-term prognosis in ulcerative colitis patients treated with vedolizumab

**DOI:** 10.1097/MD.0000000000035799

**Published:** 2023-11-03

**Authors:** Jing Yan, Ailing Liu, Liang Fang, Jun Wu, Xueli Ding, Yonghong Xu

**Affiliations:** a Department of Gastroenterology, The Affiliated Hospital of Qingdao University, Qingdao, Shandong Province, China.

**Keywords:** degree of ulcerative colitis burden of luminal inflammation, mayo endoscopic score, ulcerative colitis, ulcerative colitis endoscopic severity index, vedolizumab

## Abstract

The scoring systems commonly used to assess endoscopic disease severity of ulcerative colitis (UC) in clinical research and practice include the Mayo endoscopic score (MES), ulcerative colitis endoscopic severity index (UCEIS), and degree of ulcerative colitis burden of luminal inflammation (DUBLIN). We aimed to assess and compare the predictive efficacy of the MES, DUBLIN score and UCEIS score for prognosis in UC patients treated with vedolizumab (VDZ).

Seventy-four UC patients who treated with VDZ from September 2021 to February 2023 were retrospectively enrolled. We used the MES, DUBLIN and UCEIS score to evaluate endoscopic findings. The predictive capability of these 3 scores for surgery or therapeutic escalation was assessed using the receiver operating characteristic curve.

The mean MES, DUBLIN and UCEIS score significantly improved from 2.83 ± 0.38, 7.80 ± 1.82 and 6.24 ± 1.51 to 2.07 ± 0.88, 5.57 ± 2.68, and 3.72 ± 2.12, respectively (*P* < .001). Lower pre-therapeutic UCEIS scores were associated with favorable short-term outcomes. Importantly, the post-therapeutic UCEIS score showed the best predictive capability with an area under curve of 0.871 (95% confidence interval: 0.767–0.976), specificity of 0.654, sensitivity of 0.900, and cutoff value of 3.5. A UCEIS score of ≥ 4 after treatment was correlated with surgical operation or treatment escalation.

The UCEIS score is superior to the MES and DUBLIN score in reflecting short-term outcomes and long-term prognosis in UC patients treated with VDZ, and clinical remission could be defined as a UCEIS score ≤ 3.

## 1. Introduction

Endoscopy is a crucial tool for the assessment of disease activity and treatment outcomes in individuals with inflammatory bowel disease (IBD).^[1]^ Endoscopic remission or mucosal healing can reduce the incidence of adverse prognoses such as recurrence, surgery, and hospitalization rates in ulcerative colitis (UC), which is a new target for UC treatment.^[2]^ The scoring system commonly used to assess endoscopic disease severity in clinical research and practice is the Mayo endoscopic score (MES),^[3]^ but it has the disadvantage of poor agreement among previous studies. The ulcerative colitis endoscopic severity index (UCEIS) that was developed by Travis et al^[4]^ in 2012, covered vascular patterns, bleeding, and erosions/ulcers, and can accurately reflect disease severity and prognosis of UC patients.^[5]^ However, the MES and UCEIS score, as assessment tools for the most severe intestinal lesions, do not include the extent of disease. Disease extent has been strongly correlated with disease recurrence, intensive therapy, and colectomy in UC patients, as reported.^[6–8]^ Hence, Rowan et al^[9]^ described the degree of ulcerative colitis burden of luminal inflammation (DUBLIN) score based on the MES and disease extent (Montreal classification). However, as new scoring systems, the UCEIS and DUBLIN scores have not yet been conclusively validated.

Vedolizumab (VDZ), a human monoclonal antibody to the α4β7 integrin, is significantly more effective than placebo in inducing and maintaining clinical remission in UC patients who have received conventional therapy but still suffer from active disease.^[10]^ Saito et al^[11]^ found that endoscopic remission at week 24 was associated with a better long-term prognosis with VDZ treatment. However, the usefulness of the DUBLIN score and UCEIS score is not determined in reflecting long-term prognosis for UC patients receiving VDZ induction therapy.

Therefore, in this study, we planned to assess and compare the predictive efficacy of the MES, DUBLIN score and UCEIS score for clinical outcomes and long-term prognosis of VDZ therapy, and to identify the threshold of optimal endoscopy score for poor prognosis in UC patients after induction therapy.

## 2. Methods

### 2.1. Patients and therapy protocol

We retrospectively reviewed 85 UC patients treated with VDZ intravenous infusion at the Affiliated Hospital of Qingdao University from September 2021 to February 2023. The diagnosis of UC was made based on established standards, which included clinical, endoscopic, radiological, and pathological criteria. Inclusion criteria involved: Active UC with a clinical activity index (CAI)^[12]^ of > 4 or an MES of ≥ 2; Patients who underwent colonoscopy within 1 month prior to starting VDZ; and Patients who had completed at least 3 doses of VDZ. Exclusion criteria were: Crohn disease, unclassified IBD, intestinal tuberculosis and Bechet’s disease; Lack of colonoscopy prior to VDZ treatment; Incomplete induction treatments; and history of colon surgery. Finally, 74 patients with active UC were enrolled in our study. VDZ was administered with 300 mg at week 0, 2 and 6, and then administered per 8 weeks.

### 2.2. Clinical efficacy and endoscopic evaluation

Clinical disease activity was evaluated by experienced physicians based on the CAI proposed by Rachmilewitz,^[12]^ which included the following items: number of stools per week, bloody stools, general well-being, temperature, abdominal pain, extraintestinal manifestations, and laboratory results of erythrocyte sedimentation rate and hemoglobin (range 0–29).

We evaluated patients by endoscopy before and after VDZ treatment (range 6–54 weeks) and assessed endoscopic severity using the MES, DUBLIN score, and UCEIS score. During endoscopy, 5 experienced endoscopists who were unaware of the results used MES and UCEIS to score the severity of the visualized colon directly under endoscopy and identified the disease extension through Montreal classification [proctitis (lesion confined to the rectum); left-sided colitis (lesion does not exceed splenic flexure); total colitis (lesion exceeding splenic flexure)].^[13]^ Two trained endoscopic physicians (A.L. and L.F.) verified the MES and UCEIS score again and calculated the DUBLIN score, which was the product of the extent of disease and MES. If the disease extent was unclear, magnetic resonance imaging or computed tomography was performed to confirm it. The UCEIS score (range 0–8) is the sum of several subscores for vascular patterns (0–2), hemorrhage (0–3), and erosions and ulcerations (0–3) in the most severe portion of mucosal inflammation. Ultimately, a senior physician (Y.X.) resolved any disagreements.

### 2.3. Definition and assessment of clinical outcome

Clinical outcomes after VDZ treatment were assessed using the CAI. Clinical response was determined as a decrease in CAI ≥ 3 from baseline and an absolute score ≤ 10. Clinical remission was considered as CAI ≤ 4. All other conditions were defined as nonresponse. Endoscopic remission was considered as MES ≤ 1. The endpoint of event-free survival was regarded as the absence of a shortening of the VDZ infusion interval (4 weeks but not 6 weeks) and the absence of an addition or switch to other treatments (colectomy, corticosteroids, tofacitinib or other biological agents) but not topical or symptomatic treatments. Assessment of the predictive value of 3 endoscopic scoring systems (MES, DUBLIN score, and UCEIS score) for event-free survival was the primary endpoint. The secondary endpoint was the comparison of the forecasting potential of the above systems for short-term treatment responses.

### 2.4. Statistical analysis

Nonparametric variables were compared using the Mann–Whitney *U* test. Paired variables before and after treatment were assessed using the Wilcoxon signed-rank test. Using Spearman rank correlation coefficient, correlations between the DUBLIN score, UCEIS score, and MES were tested. The predictive capability of MES, DUBLIN and UCEIS for surgical and pharmacological escalation was assessed by the receiver operating characteristic (ROC) curve, and the optimal cutoff value was determined. We used Kaplan–Meier analysis and multiple log-rank tests to assess event-free survival rates. All statistical analyses were performed using SPSS 26.0 software (IBM, USA), and the *P* < .05 was considered as statistically significant.

### 2.5. Ethical statement

Approval for the study was obtained from the Ethics Committee of the Affiliated Hospital of Qingdao University (QYFY WZLL 27687).

## 3. Results

### 3.1. Patient characteristics at baseline

Among the 74 UC patients enrolled in our study (Table 1), 50 (67.6%) were male and 24 (32.4%) were female. The median age was 48 years with interquartile range (IQR) of 34-57 years and the median disease duration was 4.5 years (IQR 2–9 years) at the initiation of VDZ infusion.

**Table 1:** Baseline characteristics of 74 patients with UC.

Characteristic	Total (n = 74)
Male/Female, n (%)	50 (67.6)/24 (32.4)
Age, yr, median (IQR)	48 (34–57)
Duration of disease, years, median (IQR)	4.5 (2.0–9.3)
Extent of colitis, n (%)	
E3	56 (75.7)
E2	17 (23.0)
E1	1 (1.3)
Treatment prior to VDZ therapy, n (%)	
5-ASA	74 (100.0)
Corticosteroids	35 (47.3)
Immunosuppressants	7 (9.5)
Anti-TNF	10 (13.5)
Concomitant medication, n (%)	
5-ASA	67 (90.5)
Corticosteroids	24 (32.4)
CAI, median (IQR)	8 (6–10)
MES, n (%)	
MES 2	14 (18.9)
MES 3	60 (81.1)
DUBLIN score, median (IQR)	9 (6–9)
UCEIS score, median (IQR)	6 (5–8)

5-ASA = 5-aminosalicylate, CAI = clinical activity index, DUBLIN = degree of ulcerative colitis burden of luminal inflammation, E1 = proctitis, E2 = left-sided colitis, E3 = total colitis, IQR = interquartile range, MES = endoscopic Mayo score, TNF = tumor necrosis factor, UC = ulcerative colitis, UCEIS = ulcerative colitis endoscopic index of severity, VDZ = vedolizumab.

Fifty-six patients (75.7%) had pancolitis and 17 (23.0%) had left-sided colitis. Before starting VDZ, 74 (100%), 35 (47.3%), 7 (9.5%) and 10 (13.5%) patients received 5-aminosalicylates, corticosteroids, immunosuppressants and anti-TNF, respectively. Twenty-four patients (32.4%) were on corticosteroids and 67 (90.5%) were receiving 5-aminosalicylate at the time of VDZ induction therapy. The median CAI at baseline was 8 (IQR 6-10). Sixty patients (81.1%) had the MES of 3 and the remaining 14 (18.9%) had the MES of 2. The median UCEIS and DUBLIN scores at baseline were 6 (IQR 5–8) and 9 (IQR 6–9), respectively.

### 3.2. Clinical efficacy

**Figure 1.**
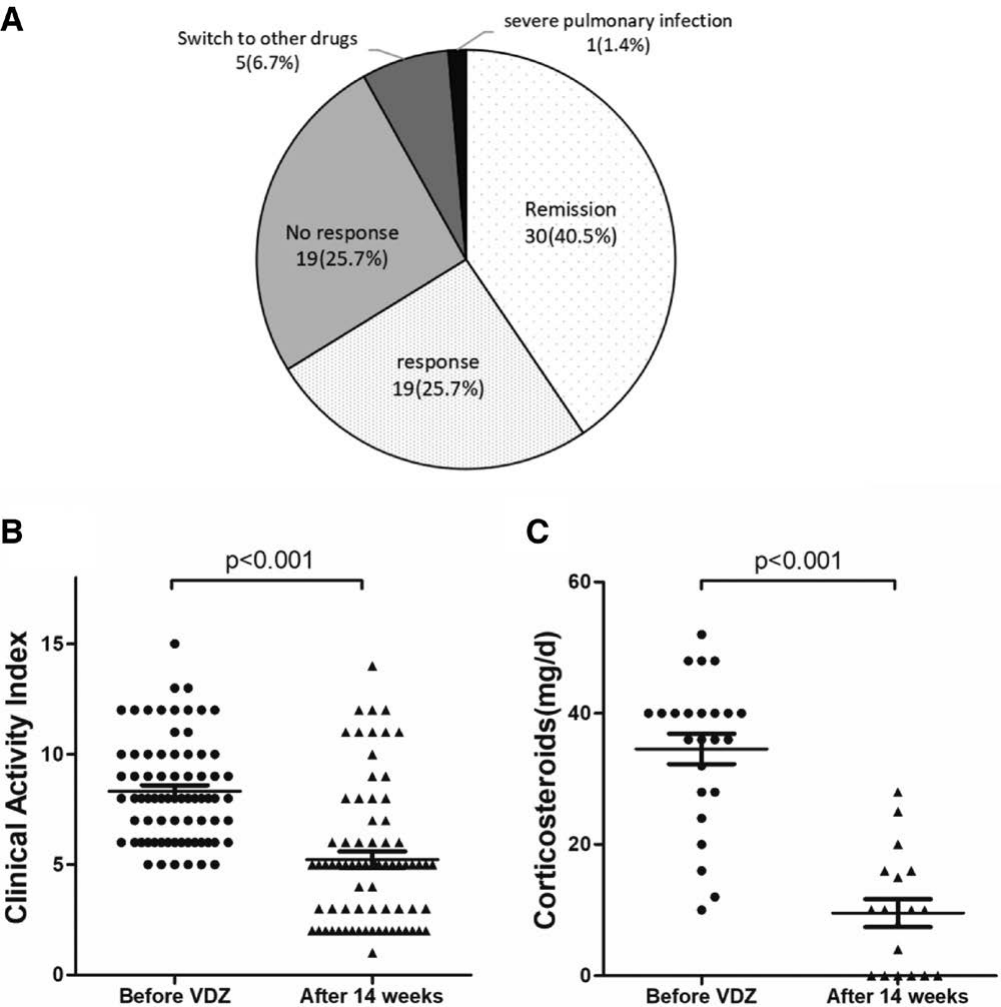
Short-term clinical outcomes of VDZ induction therapy. (A) Clinical responses were assessed using CAI at 14 weeks. Remission was defined as a CAI ≤ 4, and response was defined as a decrease in CAI from baseline of at least 3 points and an absolute score ≤ 10. (B) Comparison of the CAI scores before and after VDZ induction therapy (n = 74). (C) Comparison of corticosteroid doses before and after VDZ induction therapy (n = 24). CAI = clinical activity index, VDZ = vedolizumab.

Figure 1 shows the short-term efficacy of VDZ induction therapy. At 14 weeks after VDZ treatment, 30 patients (40.5%) achieved clinical remission and 19 patients (25.7%) achieved clinical response. Five patients (6.7%) needed to switch to other therapies and 1 (1.4%) discontinued VDZ due to severe pulmonary infection (Fig. 1A). The mean CAI score decreased from 8.32 at baseline to 5.32 at week 14 (*P* < .001) (Fig. 1B). Corticosteroid therapy was discontinued in 6 of the 24 patients (25.0%), and the mean steroid dose was reduced from 34.58 to 9.56 mg/day at week 14 in these 18 patients excluding 6 patients for whom steroid dose data were missing (*P* < .001) (Fig. 1C). None of the patients underwent colectomy during the follow-up period.

### 3.3. Endoscopic results

**Figure 2.**
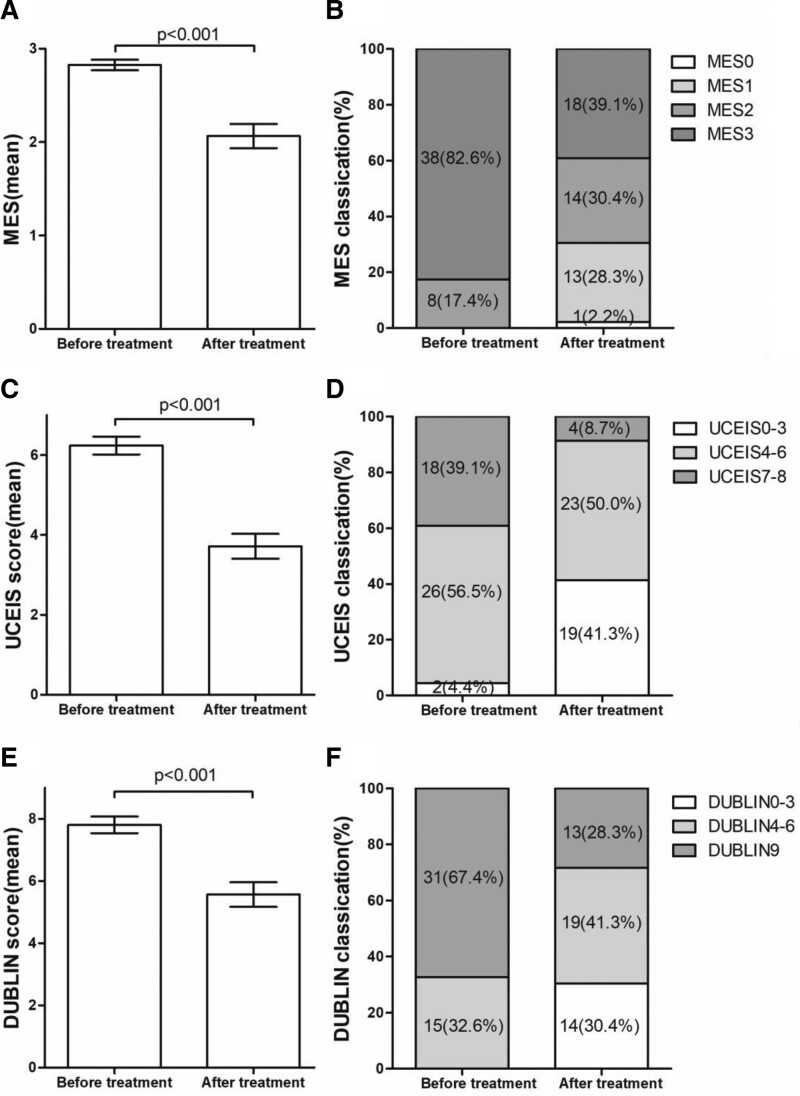
Endoscopic improvement with VDZ induction therapy (n = 46). (A–B) Comparison of the MES before and after VDZ induction therapy. (C–D) Comparison of the UCEIS score before and after VDZ induction therapy. (E–F) Comparison of the DUBLIN score before and after VDZ induction therapy. DUBLIN = degree of ulcerative colitis burden of luminal inflammation, MES = Mayo endoscopic score, UCEIS = ulcerative colitis endoscopic severity index, VDZ = vedolizumab.

Improvement in endoscopy was assessed using the MES, UCEIS score and DUBLIN score in 46 patients undergoing endoscopy 6 to 54 weeks after initiation of VDZ. Changes in the mean score and classification before and after VDZ treatment are shown in Figure 2. The mean MES significantly improved from 2.83 ± 0.38 (with a score of 2 in 8 patients [17.4%] and 3 in 38 [82.6%]) to 2.07 ± 0.88 (with a score of 0 in 1 patient [2.2%], 1 in 13 [28.3%], 2 in 14 [30.4%], and 3 in 18 [39.1%]) (*P* < .001) (Fig. 2A and B). The mean UCEIS significantly decreased from 6.24 ± 1.51 (with a score of 0–3 in 2 patients [4.4%], 4–6 in 26 [56.5%], and 7–8 in 18 [39.1%]) to 3.72 ± 2.12 (with a score of 0–3 in 19 patients [41.3%], 4–6 in 23 [50.0%], and 7–8 in 4 [8.7%]) (*P* < .001) (Fig. 2C and D). And DUBLIN improved significantly from a mean of 7.80 ± 1.82 (with a score of 4–6 in 15 patients [32.6%] and 9 in 31 [67.4%]) to 5.57 ± 2.68 (with a score of 0–3 in 14 [30.4%], 4–6 in 19 [41.3%], and 9 in 13 [28.3%]) (*P* < .001) (Fig. 2E and F).

**Figure 3.**
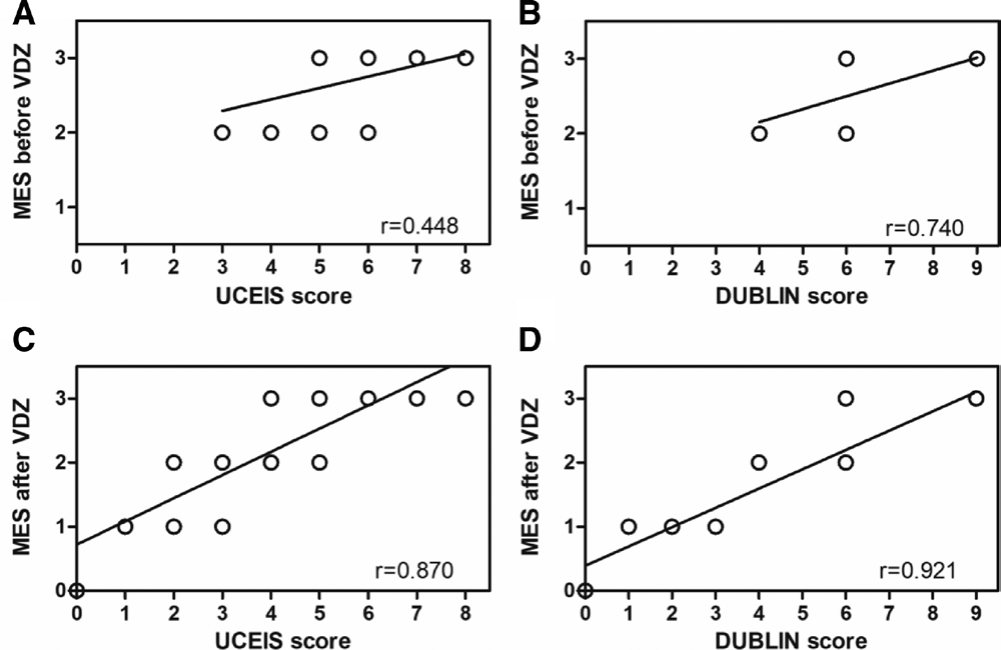
Correlations between the MES, UCEIS, and DUBLIN score before and after VDZ treatment. (A) A weak correlation between the pre-therapeutic MES and UCEIS score (*R* = 0.448, *P* = .002). (B) A strong correlation between the pre-therapeutic MES and DUBLIN score (*R* = 0.740, *P* < .001). (C) A strong correlation between the post-therapeutic MES and UCEIS score (*R* = 0.870, *P* < .001). (D) A strong correlation between the post-therapeutic MES and DUBLIN score (*R* = 0.921, *P* < .001). DUBLIN = degree of ulcerative colitis burden of luminal inflammation, MES = Mayo endoscopic score, UCEIS = ulcerative colitis endoscopic severity index, VDZ = vedolizumab.

Correlations between the MES, DUBLIN score, and UCEIS score before and after VDZ therapy were tested, as shown in the scatter plot in Figure 3. Pre-therapeutic UCEIS scores were weakly associated with MES (*R* = 0.448, *P* = .002; Fig. 3A), whereas the association between pre-therapeutic DUBLIN scores and MES was strong (*R* = 0.740, *P* < .001; Fig. 3B). Similarly, both the post-therapeutic UCEIS and DUBLIN scores were strongly correlated with MES (*R* = 0.870, *P* < .001; Fig. 3C and *R* = 0.921, *P* < .001; Fig. 3D). The MES 3 corresponded to a wide range of UCEIS scores (5–8 pretreatment and 4–8 posttreatment) and DUBLIN scores (6–9 both pre- and posttreatment), indicating that UCEIS and DUBLIN scores are more quantitative than MES for patients with severe UC.

### 3.4. Comparison of pre-therapeutic MES, DUBLIN and UCEIS in predicting short-term clinical remission and endoscopic remission

**Figure 4.**
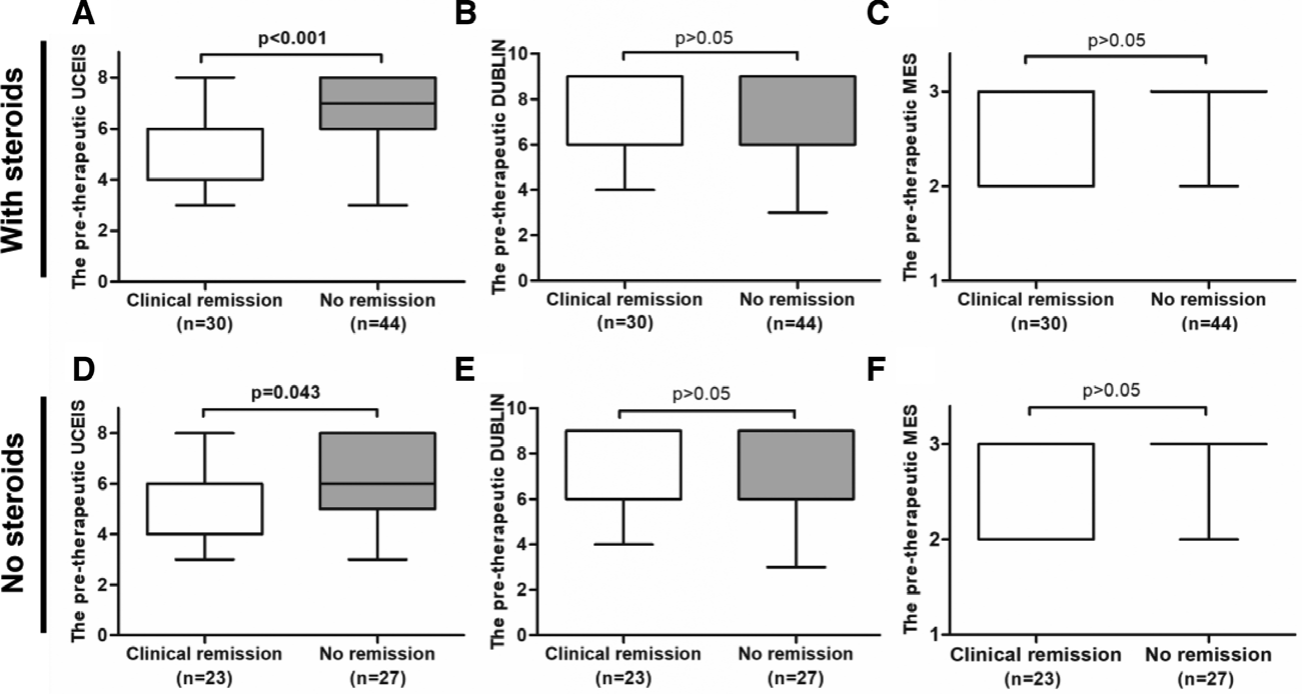
Comparison of the pre-therapeutic UCEIS, DUBLIN, and MES between the clinical remission and non-remission groups at 14 weeks of VDZ therapy. (A) The pre-therapeutic UCEIS score was significantly lower in the group that reached clinical remission than in those that did not reach clinical remission at 14 weeks of VDZ therapy (*P* < .001). (B) No significant difference in correlation between pre-therapeutic DUBLIN score and clinical remission at 14 weeks (*P* > .05). (C) No significant difference in correlation between pre-therapeutic MES and clinical remission at 14 weeks (*P* > .05). (D) For 50 patients treated with VDZ but not in combination with steroids, the pre-therapeutic UCEIS score was significantly lower in the group that reached clinical remission than in those that did not reach clinical remission. (*P* = .043). (E) No significant difference in correlation between pre-therapeutic DUBLIN score and clinical remission at 14 weeks for patients treated with VDZ but not in combination with steroids (*P* > .05). (F) No significant difference in correlation between pre-therapeutic MES and clinical remission at 14 weeks for patients treated with VDZ but not in combination with steroids (*P* > .05). DUBLIN = degree of ulcerative colitis burden of luminal inflammation, MES = Mayo endoscopic score, UCEIS = ulcerative colitis endoscopic severity index, VDZ = vedolizumab.

Of the 74 patients at 14 weeks after VDZ therapy, those who achieved clinical remission had significantly lower pre-therapeutic UCEIS scores than those who did not achieve clinical remission (*P* < .001) (Fig. 4A), while there was no significant difference in the pre-therapeutic MES and DUBLIN scores between clinical remission and non-remission groups (*P* > .05) (Fig. 4B and C). We observed similar results in patients treated with VDZ but not in combination with steroids (Fig. 4D and F).

Similarly, in an analysis of 46 patients undergoing endoscopy after the initiation of VDZ, those with endoscopic remission had significantly lower pre-therapeutic UCEIS scores than those with endoscopic non-remission (*P* = .002) (Figure S1 A, Supplemental Digital Content, http://links.lww.com/MD/K487), however, pre-therapeutic MES and DUBLIN scores did not differ between the 2 groups (*P* > .05) (Figure S1 B and C, Supplemental Digital Content, http://links.lww.com/MD/K487). This suggests that the pre-therapeutic UCEIS score may be superior to the MES and DUBLIN score in predicting short-term response to VDZ treatment in patients with UC.

### 3.5. Comparison of post-therapeutic MES, DUBLIN and UCEIS in predicting long-term prognosis

**Figure 5.**
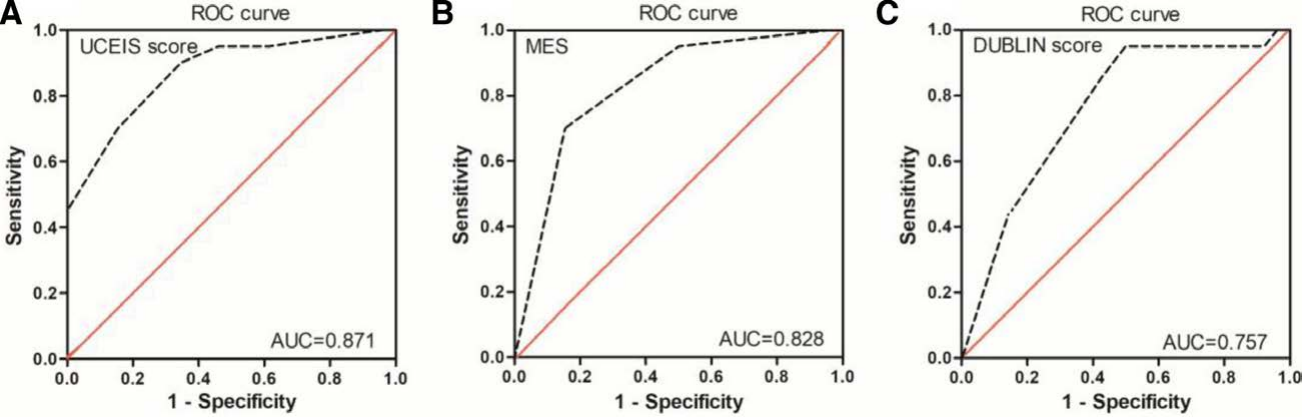
ROC curves of the MES versus UCEIS score versus DUBLIN score in predicting long-term event-free survival of UC patients treated with VDZ (n = 46). (A) The AUC of the UCEIS score (AUC = 0.871). (B) The AUC of the MES (AUC = 0.828). (C) The AUC of the DUBLIN score (AUC = 0.757). AUC = area under the curve, DUBLIN = degree of ulcerative colitis burden of luminal inflammation, MES = Mayo endoscopic score, ROC = receiver operating characteristic, UC = ulcerative colitis, UCEIS = ulcerative colitis endoscopic severity index, VDZ = vedolizumab.

**Figure 6.**
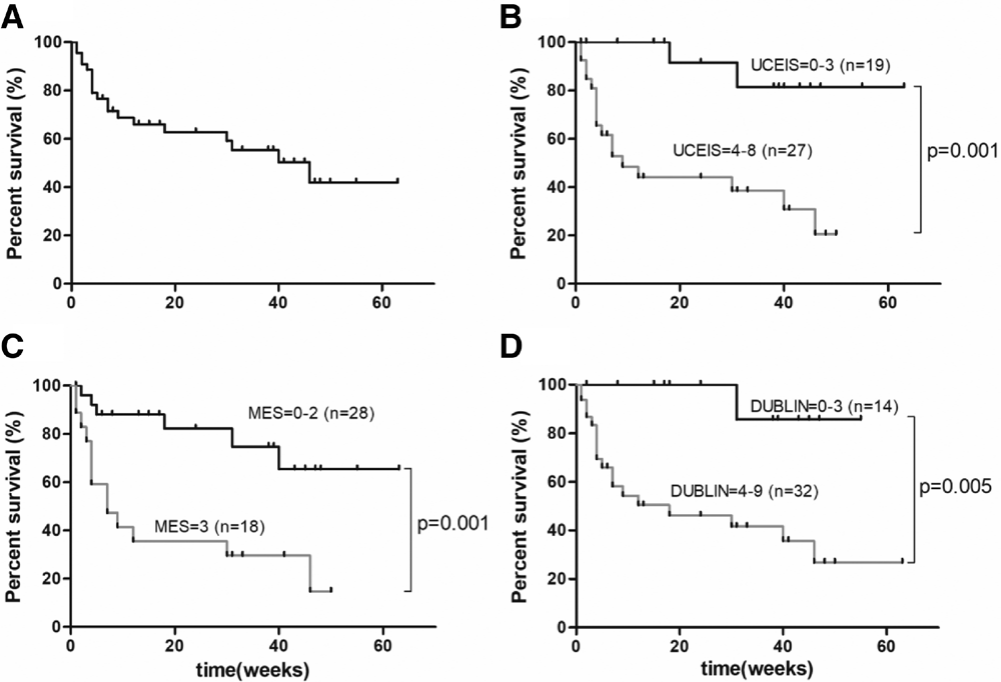
Long-term prognosis of patients treated with VDZ. (A) Kaplan–Meier analysis revealed event-free survival in 46 patients treated with VDZ. The event-free survival rate was defined as the percentage of patients who did not require surgery or other pharmacological therapy. (B) The event-free survival rates of patients with the UCEIS score of 0–3 and those with the UCEIS score of 4–8. (C) The event-free survival rates of patients with the MES of 0–2 and those with the MES of 3. (D) The event-free survival rates of patients with the DUBLIN score of 0–3 and those with the DUBLIN score of 4–9. DUBLIN = degree of ulcerative colitis burden of luminal inflammation, MES = Mayo endoscopic score, UCEIS = ulcerative colitis endoscopic severity index, VDZ = vedolizumab,.

The event-free survival rates after post-therapeutic colonoscopy (average interval time, 26 weeks) are shown in Figure 6. In 46 UC patients undergoing post-therapeutic colonoscopy, the event-free survival rates were 71.1% at 24 weeks and 48.7% at 54 weeks (Fig. 6A). The accuracy of the endoscopic scores (MES, DUBLIN and UCEIS score) in predicting long-term survival was assessed and compared using ROC curve analysis. The post-therapeutic UCEIS score showed the best predictive capability with an area under the curve (AUC) of 0.871 (95% confidence interval [CI]:0.767–0.976), specificity of 0.654, sensitivity of 0.900, and cutoff value of 3.5 (Fig. 5A). Therefore, the UC patients were categorized into UCEIS ≤ 3 (n = 19) and UCEIS ≥ 4 (n = 27). A significantly higher event-free survival rate was observed in patients with UCEIS scores ≤ 3 than in those with UCEIS scores ≥ 4 group (*P* = .001) (Fig. 6B). However, the post-therapeutic MES, with an AUC of 0.828 (95% CI:0.706–0.949), specificity of 0.846, sensitivity of 0.700, and cutoff value of 2.5, had lower predictive power than the UCEIS score (Fig. 5B). Patients who achieved MES ≤ 2 (n = 28) exhibited significantly better event-free survival rates than those with MES of 3 (n = 18) (*P* = .001) (Fig. 6C). Compared with the UCEIS score and MES, the DUBLIN score showed inferior predictive power with an AUC of 0.757 (95% CI: 0.614–0.899), specificity of 0.500, sensitivity of 0.950, and cutoff value of 3.5 (Fig. 5C). There was a significantly higher rate of event-free survival in patients with DUBLIN scores ≤ 3 (n = 14) than in those with DUBLIN scores ≥ 4 (n = 32) (*P* = .005) (Fig. 6D).

Therefore, the post-therapeutic UCEIS score was superior to the MES and DUBLIN score in predicting surgical and pharmacological escalation of UC patients treated with VDZ therapy. The rate of cumulative event-free survival for patients with UCEIS ≤ 3 was 100.0% at 24 weeks and 88.9% at 54 weeks. In comparison, the cumulative overall event-free survival was 53.8% at 24 weeks and 30.7% at 54 weeks in the UCEIS ≥ 4 group (Fig. 6B). The correlation of long-term prognosis with the UCEIS score, stratifying the patients on the basis of endoscopic assessment intervals (before 24 weeks vs after 24 weeks), was also analyzed. There was no difference in the association of the UCEIS score with event-free survival regardless of the duration of colonoscopic assessment (*P* = .934) (Figure S2, Supplemental Digital Content, http://links.lww.com/MD/K488).

## 4. Discussion

In this study, the predictive potential of MES, DUBLIN and UCEIS in UC patients receiving VDZ was evaluated and compared. Of note, the UCEIS has shown better capability than MES and DUBLIN in predicting short-term clinical responses and long-term outcomes. The pre-therapeutic UCEIS score was correlated with clinical remission at week 14 and endoscopic remission after VDZ treatment. In addition, the post-therapeutic UCEIS score was better than the MES and DUBLIN score in reflecting long-term prognosis of UC patients treated with VDZ.

Endoscopic remission has now become a key therapeutic goal in the administration of UC,^[2]^ and endoscopy has a critical function in reflecting inflammation of the intestinal mucosa and therapeutic efficacy in patients with IBD.^[3]^ To date, several endoscopic scoring systems have been established for the administration of UC, such as the MES,^[3]^ Baron,^[14]^ Rachmilewitz,^[15]^ UCEIS,^[4]^ and DUBLIN^[9]^ scores, but most of these have not been validated. Although the MES is the most frequently used scoring system for endoscopic evaluation in clinical settings, Di Ruscio^[16]^ and Xie et al^[17]^ proved that UCEIS exhibits superior predictive value to MES in predicting the response to immunomodulatory agents or biologic therapy, and the need for colectomy in patients with UC. The DUBLIN score, proposed by Rowan et al^[9]^, considers the significance of disease location in the treatment and management of UC to assess endoscopic activity in conjunction with MES. Recently, Zhang et al^[18]^ compared the baseline MES, DUBLIN and UCEIS scores for clinical application, demonstrating that the UCEIS score is more valuable in reflecting the clinical outcomes of UC patients treated with steroid therapy, whereas the DUBLIN score could better predict the clinical severity of UC. Only 3.9% of patients in their study received biological therapy. However, previous studies have suggested that the optimal threshold of UCEIS and DUBLIN associated with long-term prognosis would differ depending on the type of conventional drug or biologic agent.^[17–19]^ In addition, the UCEIS value that defines mucosal healing or endoscopic remission remains controversial.^[20,21]^ Here, we first compared the predictive efficacy of MES, DUBLIN and UCEIS scores for short-term response and long-term prognosis of VDZ treatment, and demonstrated the optimal UCEIS and DUBLIN thresholds associated with the requirement of surgery or other pharmacological treatment after induction therapy.

In our study, the MES, UCEIS score and DUBLIN score all improved after VDZ therapy. In the comparison between the UCEIS score, DUBLIN score, and MES, similar to the study of Zhang et al^[18]^, the UCEIS and DUBLIN scores ranged considerably within each of the MES classifications, reflecting their different assessment values: the UCEIS score assesses disease activity of the most severe mucosa of the intestine in terms of a combination of vascular patterns, bleeding, and erosions/ulcers;^[4]^ the DUBLIN score considers both disease extent and endoscopic severity;^[9]^ whereas the MES does not distinguish between deep and superficial ulcers and lacks an accurate description of bleeding.^[5]^ Therefore, we think that the UCEIS score and DUBLIN score are more quantitative and objective than the MES in reflecting clinical outcome after VDZ treatment.

Recently, Di Ruscio et al^[16]^ found that high baseline UCEIS scores were related to loss of response to biologics and the need for colon resection in patients with UC. But only 3 UC patients in their study were administered with VDZ. In our study, the post-therapeutic UCEIS score provided superior predictive value for treatment escalation in UC patients undergoing VDZ therapy than MES and DUBLIN score. Although all 3 scoring systems differed significantly in predicting event-free survival after VDZ treatment, the post-therapeutic UCEIS score based on ROC analysis showed the highest AUC (0.827). A significantly higher event-free survival rate was observed in patients with UCEIS score ≤ 3, and most patients in the UCEIS ≥ 4 group discontinued VDZ or added or changed to other medications during the observation period, with results close to those reported by de Jong et al^[22]^. In their study, 94% patients with a UCEIS ≥ 4 required treatment optimization or escalation. Notably, de Jong et al^[22]^ and Di Ruscio et al^[16]^ focused on the association between the pre-therapeutic UCEIS score and prognosis. However, VDZ, as a targeted agent, has a long onset of action.^[23]^ Therefore, regarding the long-term prognosis of UC patients, the mucosal status after therapy seems to have more predictive value than before therapy. In addition, similar to Di Ruscio study,^[16]^ we found that pre-therapeutic UCEIS score, but not MES and DUBLIN score, was significantly associated with short-term clinical and endoscopic remission after VDZ therapy. The UCEIS accurately evaluates the condition of the colonic mucosa from 3 aspects (vascular patterns, bleeding, and erosions or ulcers), which includes more details than MES and DUBLIN score, probably leading to the above results.

However, the optimal UCEIS cutoff value correlated with poor outcome and prognosis for UC patients treated with VDZ therapy remains undefined.^[20,21]^ Saigusa et al^[20]^ showed that patients with a UCEIS ≤ 1 after infliximab treatment had a higher long-term event-free survival rate and concluded that mucosal healing could be identified as a UCEIS ≤ 1. Additionally, Ikeya et al^[5]^ built on this definition by limiting “the UCEIS score 1 to a vascular pattern descriptor”. In a recent prospective study, Golovics et al^[21]^ found that a UCEIS score ≤ 3 was defined as the best threshold for clinical or endoscopic remission, whereas a UCEIS score ≥ 4 was identified as an active lesion. Similarly, we found that MES ≤ 1 corresponded to a UCEIS score range of 0 to 3, and most UC patients treated with VDZ in the post-therapeutic UCEIS ≥ 4 group required surgery or other pharmacological treatments during the follow-up period. Although the threshold for mucosal healing is identified as UCEIS ≤ 1, clinical remission may be defined as a UCEIS score ≤ 3. Furthermore, the cutoff value of a post-therapeutic UCEIS score ≥ 4 could indicate that surgery or escalation therapy is a more reasonable option for UC patients after VDZ induction treatment.

There are some limitations in this study. First, in this retrospective study, post-therapeutic colonoscopies were not planned at a fixed time, and the time interval between the pre- and post-therapeutic endoscopies varied widely (6–54 weeks). However, no differences in the correlation between UCEIS scores and long-term prognosis were found regardless of the duration of colonoscopy assessment. Second, still images of colonoscopy were used in our study to evaluate the UCEIS scores, which were initially developed and validated according to sigmoidoscopy videos,^[24]^ but the UCEIS scores calculated by applying still images had similar accuracy to that of videos.^[25]^ Third, only 74 patients from a single medical center were enrolled in this study, and we will conduct a prospective multicenter study to confirm our findings in the future.

In summary, we found that the UCEIS score was superior to the MES and DUBLIN score in assessing disease activity and predicting short-term outcomes and long-term prognosis in UC patients treated with VDZ. Furthermore, a UCEIS score of ≥ 4 after VDZ treatment was relevant to the need for surgery and therapeutic escalation in patients. Therefore, UCEIS is a useful tool for the management of patients with UC.

## Author contributions

**Conceptualization:** Jing Yan.

**Data curation:** Ailing Liu, Liang Fang.

**Formal analysis:** Jing Yan, Jun Wu.

**Investigation:** Liang Fang, Jun Wu, Yonghong Xu.

**Methodology:** Ailing Liu, Xueli Ding, Yonghong Xu.

**Project administration:** Xueli Ding.

**Resources:** Jun Wu.

**Software:** Xueli Ding.

**Supervision:** Xueli Ding, Yonghong Xu.

**Validation:** Yonghong Xu.

**Visualization:** Jing Yan, Jun Wu.

**Writing – original draft:** Jing Yan, Ailing Liu, Liang Fang.

**Writing – review & editing:** Yonghong Xu.

## Supplementary Material
