# Supplementary material for: The ulcerative colitis endoscopic index of severity score is superior to reflecting long-term prognosis in ulcerative colitis patients treated with vedolizumab
Source: Medicine (Baltimore). 2023 Nov 3;102(44):e35799. doi: 10.1097/MD.0000000000035799 (PMC10627604; doi:10.1097/MD.0000000000035799)
Supplement: Supplementary file 1 [file medi-102-e35799-s001.docx]

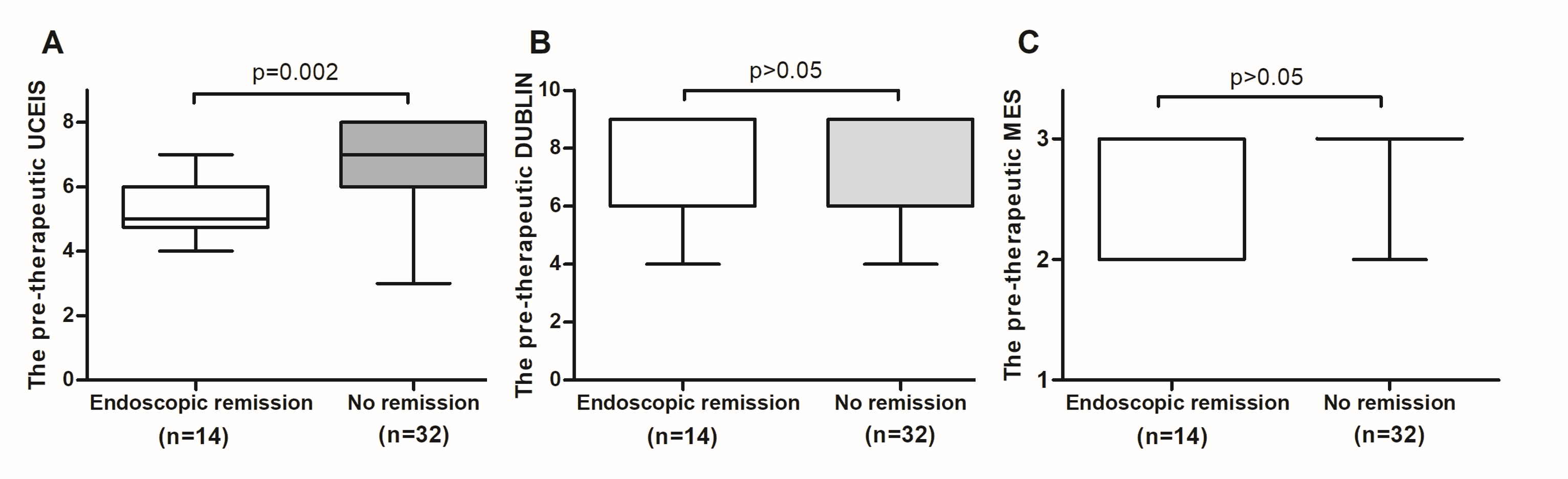
Supplementary Fig. 1 Comparison of the pre-therapeutic UCEIS, DUBLIN and MES between the endoscopic remission and non-remission groups in patients who underwent colonoscopy after VDZ therapy (n=46). (A) The pre-therapeutic UCEIS score was significantly lower in the endoscopic remission group than non-remission group (p=0.002). (B) No significant difference in correlation between pre-therapeutic DUBLIN score and endoscopic remission (p>0.05). (C) No significant difference in correlation between pre-therapeutic MES and endoscopic remission (p>0.05). VDZ=vedolizumab, MES=Mayo endoscopic score, UCEIS=ulcerative colitis endoscopic severity index, DUBLIN=degree of ulcerative colitis burden of luminal inflammation.
