# Supplementary material for: The ulcerative colitis endoscopic index of severity score is superior to reflecting long-term prognosis in ulcerative colitis patients treated with vedolizumab
Source: Medicine (Baltimore). 2023 Nov 3;102(44):e35799. doi: 10.1097/MD.0000000000035799 (PMC10627604; doi:10.1097/MD.0000000000035799)
Supplement: Supplementary file 2 [file medi-102-e35799-s002.docx]

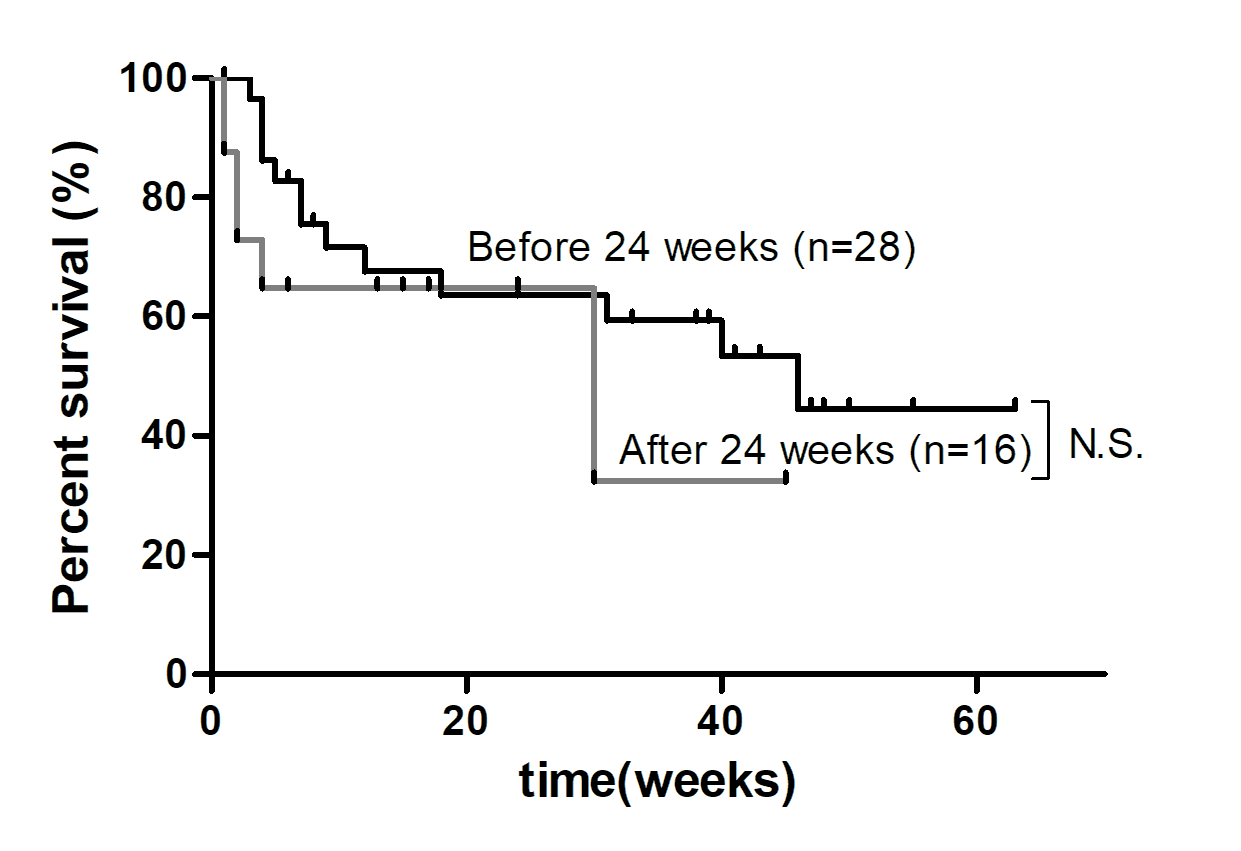


Supplementary Fig. 2 Long-term prognosis was analyzed in patients according to endoscopic assessment intervals (before 24 weeks vs. after 24 weeks).
